# Ultralow threading dislocation density in GaN epilayer on near-strain-free GaN compliant buffer layer and its applications in hetero-epitaxial LEDs

**DOI:** 10.1038/srep13671

**Published:** 2015-09-02

**Authors:** Huan-Yu Shih, Makoto Shiojiri, Ching-Hsiang Chen, Sheng-Fu Yu, Chung-Ting Ko, Jer-Ren Yang, Ray-Ming Lin, Miin-Jang Chen

**Affiliations:** 1 Department of Materials Science and Engineering, National Taiwan University, Taipei, Taiwan; 2 Kyoto Institute of Technology, Kyoto, Japan; 3 Graduate Institute of Applied Science & Technology, National Taiwan University of Science & Technology, Taipei, Taiwan; 4 Department of Electronic Engineering, Chang Gung University, Taoyuan, Taiwan

## Abstract

High threading dislocation (TD) density in GaN-based devices is a long unresolved problem because of the large lattice mismatch between GaN and the substrate, which causes a major obstacle for the further improvement of next-generation high-efficiency solid-state lighting and high-power electronics. Here, we report InGaN/GaN LEDs with ultralow TD density and improved efficiency on a sapphire substrate, on which a near strain-free GaN compliant buffer layer was grown by remote plasma atomic layer deposition. This “compliant” buffer layer is capable of relaxing strain due to the absorption of misfit dislocations in a region within ~10 nm from the interface, leading to a high-quality overlying GaN epilayer with an unusual TD density as low as 2.2 × 10^5^ cm^−2^. In addition, this GaN compliant buffer layer exhibits excellent uniformity up to a 6” wafer, revealing a promising means to realize large-area GaN hetero-epitaxy for efficient LEDs and high-power transistors.

High-efficiency solid-state lighting and high-power electronics are considered one of the most important innovations for next-generation energy-saving devices. GaN-based materials have attracted considerable attention due to their distinct properties, including a wide direct bandgap, high chemical and thermal stability, and high mobility, which makes them useful for efficient LEDs^1^,^2^,^3^,^4^ and high electron mobility transistors (HEMTs)^5^. However, due to the lack of bulk GaN substrate, GaN-based materials are conventionally grown by metal-organic chemical vapor deposition (MOCVD) on lattice mismatched substrates, such as sapphire, SiC, and Si, assisted with a low-temperature GaN or AlN nucleation layer (NL). Such hetero-epitaxial growth on lattice-mismatched substrates typically results in a high TD density (10^7^–10^10^ cm^2^) because of the large lattice mismatch and difference in thermal expansion coefficients. This high defect density would give rise to numerous nonradiative recombination sites and scattering centers, deteriorating the electrical and optical characteristics of GaN-based devices. V-shaped defects or inverted hexagonal pyramid defects in InGaN/GaN multiple quantum wells (MQWs) generally nucleate on the TDs across the first InGaN QW layer. This structure was predicted by Wu *et al.*^6^ and confirmed by Shiojiri *et al.*^7^,^8^. The V-shaped defect decorating every TD prevents the carriers from entering and recombining nonradiatively at the TD. This property is one reason why bright InGaN/GaN blue and green LEDs can be produced despite the presence of numerous TDs^3^,^4^. However, some of the carriers recombine radiatively at small MQWs enclosing the V-shaped defect, which emits light at a wavelength different from that of the main peak^6^,^7^,^8^,^9^. Moreover, the effective volume contributing to the main emission is reduced by the V-shaped defects. Hence, the light emission efficiency of InGaN/GaN LEDs might increase if the TD density can be suppressed. In addition, the efficiency of ultraviolet GaN-based LEDs is more sensitive to the defects in epitaxial layers than that of the green and blue LEDs, which is attributed to the lack of localized states in MQWs^10^,^11^,^12^. Furthermore, GaN with a low TD density  < 10^6^ cm^2^ is also highly desirable because it can increase the lifetime for continuous operation of laser diodes. Obviously, the improvement of hetero-epitaxial GaN quality to achieve a lower TD density is a crucial issue.

Many efforts have been made to reduce TD density. The most promising technique that has been developed is epitaxial lateral overgrowth (ELOG), which allows the TD density to be effectively reduced to 10^6^–10^7^ cm^−2^ 13,14. However, such improvement comes at the expense of a dramatic increase in cost due to the complicated lithography and re-growth involved in the ELOG process and the induced inhomogeneous stress distribution^15^. Atomic layer deposition (ALD) is an attractive technique for the growth of high-quality GaN; in this method, the films are prepared using layer-by-layer and self-limiting growth^16^,^17^,^18^,^19^,^20^,^21^. Recently, Ozgit-Akgun *et al.* demonstrated the growth and applications of GaN, AlN and Al_x_Ga_1-x_N thin films prepared by hollow cathode plasma-assisted ALD^22^,^23^,^24^. Compared with other thin-film deposition techniques, ALD provides unique benefits including accurate thickness and composition control with one monolayer accuracy, high uniformity over a large area, excellent conformality and step coverage on very-high-aspect-ratio structures, low defect density, and nanolaminate engineering^13^,^14^,^15^,^16^,^17^.

In this paper, a GaN compliant buffer layer (BL) grown by remote plasma ALD (RP-ALD) and treated by post-deposition annealing (PDA) on a sapphire substrate was used to substitute the conventional GaN NL prepared by MOCVD. This GaN BL plays a “compliant” role by absorbing the misfit dislocations generated by the lattice mismatch between GaN and the sapphire substrate, which also results in relaxation of the strain caused by the hetero-epitaxial misfit. Accordingly, the overlying GaN epilayer grown on this GaN compliant BL exhibits a suppressed TD density, as low as 2.2 × 10^5^ cm^−2^, which is much lower than the high TD densities obtained by conventional MOCVD GaN hetero-epitaxy (10^7^–10^10^ cm^2^) and the ELOG technique (10^6^–10^7^ cm^−2^). This ultralow TD density contributes to high-performance InGaN/GaN LEDs with a higher light emission efficiency compared with those grown on a conventional MOCVD NL.

## Results and Discussion

### Characteristics of the ALD GaN compliant buffer layer (BL)

Figure S2 in the Supplementary Information shows the GaN growth rate per ALD cycle as a function of the TEG pulse time. The growth rate increases with the TEG dose and saturates at 0.077 nm/cycle, when the TEG pulse time is greater than 0.5 sec, suggesting that the GaN thin films were grown with self-limiting characteristics by the RP-ALD technique. Figure S3 in the Supplementary Information illustrates the uniformity of the pseudo-refractive index and the film thickness of the as-deposited GaN BL on the sapphire substrate with an effective area corresponding to a 6” diameter. The percent non-uniformity values of the pseudo-refractive index and film thickness for the as-deposited GaN BL are only ±1.97% and ±0.54%, respectively, indicating high uniformity of the GaN layer prepared by PR-ALD. This wafer-scale uniformity originates from the self-limiting growth because of insensitivity to the inhomogeneous temperature and precursor distributions in the ALD reactor. The result clearly demonstrates that the ALD technique has an excellent ability to grow nitride BL with high accuracy and uniformity over a large area.

**Figure 1:**
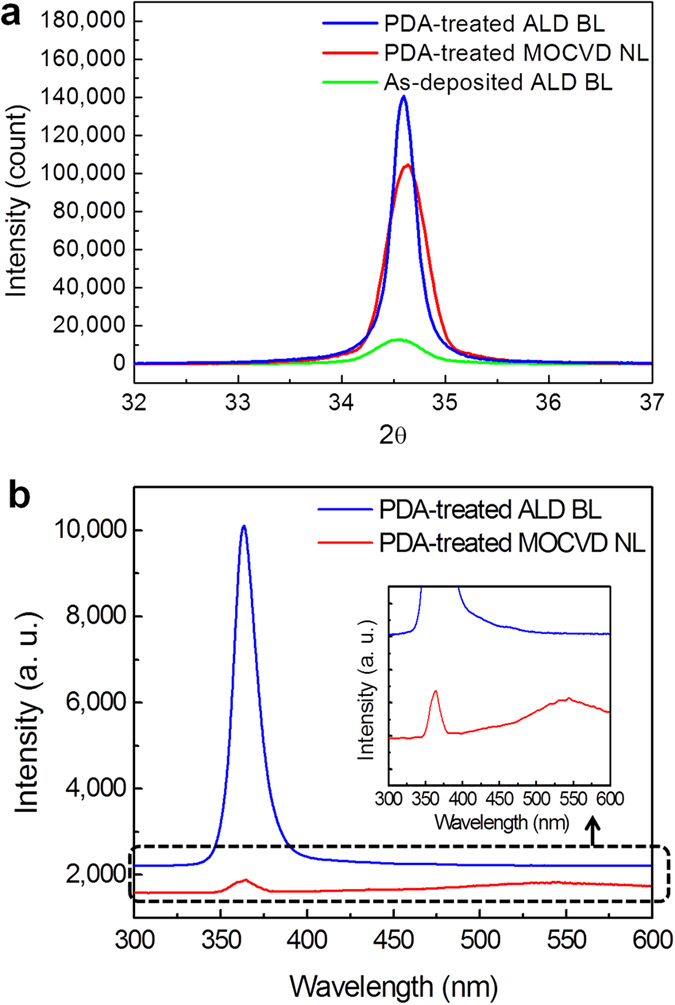
Characteristics of the ALD GaN buffer layer. (**a**) XRD patterns of the as-deposited and PDA-treated GaN BLs grown by RP-ALD together with that of the PDA-treated GaN NL grown by MOCVD. (**b**) Room-temperature micro-PL spectra of the PDA-treated ALD BL and the PDA-treated MOCVD NL. The magnified PL spectra are shown in the inset.

The X-ray diffraction (XRD) patterns of as-deposited and PDA-treated GaN BLs grown by RP-ALD are shown in Fig. 1(a), with a comparison to the PDA-treated GaN NL grown by MOCVD. Significant reflection at 34.6° is associated with the hexagonal GaN (0002), indicating that both the as-deposited and PDA-treated GaN BLs were crystallized with a wurtzite structure. The remarkable enhancement of the XRD peak intensity (green and blue lines) reveals that the PDA treatment greatly improves the crystallinity of the GaN BL grown by RP-ALD. Accordingly, it can be deduced that the as-deposited GaN is composed of fine grains and thus exhibits the diffuse XRD peak. During the PDA treatment, the fine grains grew by recrystallization and coalescence and with each other, resulting in a nearly perfect single crystal exhibiting the strong (0002) reflection peak in the XRD pattern. Notably, the XRD peak intensity and full width at half maximum (FWHM) of the PDA-treated ALD BL are superior to those of the PDA-treated MOCVD NL, indicating better crystal quality in the GaN layer grown by RP-ALD. The room-temperature micro-photoluminescence (PL) spectra of the PDA-treated ALD BL and the PDA-treated MOCVD NL are shown in Fig. 1(b). The near-band-edge PL intensity at 364 nm of the ALD BL is significantly higher than that of the MOCVD NL, and the defect-related band at approximately 550 nm is much weaker, which also indicates the superior crystal quality of the ALD BL.

**Figure 2:**
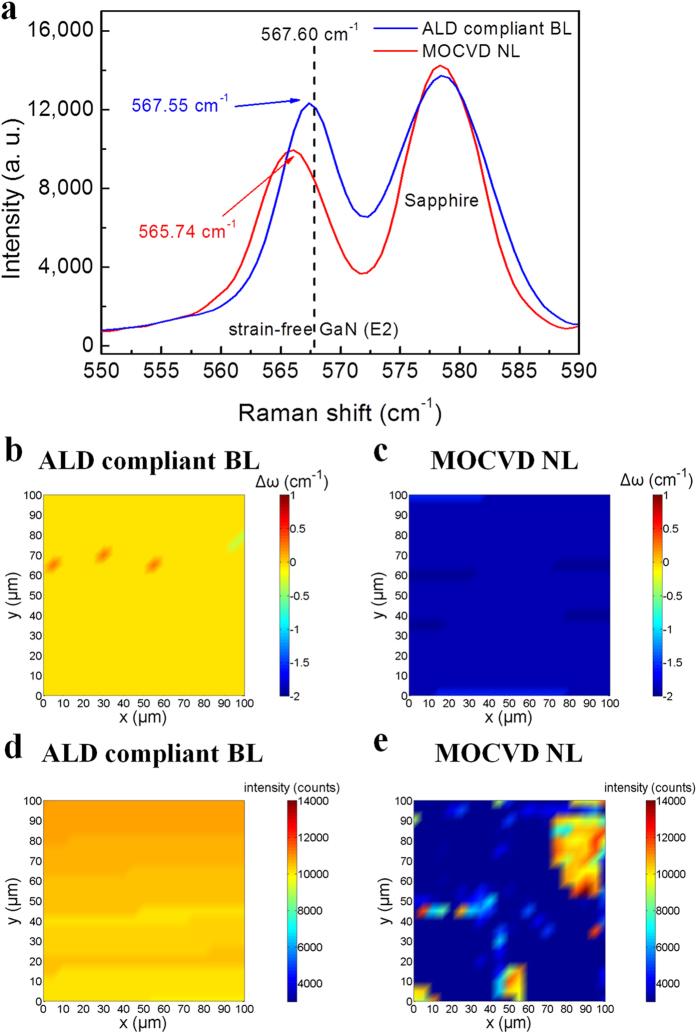
Raman analysis. (**a**) Micro-Raman spectra of the ALD compliant BL and MOCVD NL. The Raman peak at 578 cm^−1^ is ascribed to the sapphire substrate. The dashed line indicates the strain-free GaN E2 peak at 567.60 cm^−1^. (**b**–**e**) Two-dimensional micro-Raman mappings of the E2 peak in an area of 100 μm × 100 μm. (**b**) and (**c**) show the Raman shift relative to the strain-free GaN E2 peak (Δω), and (**d**) and (**e**) show the Raman intensity, of the ALD compliant BL and MOCVD NL, respectively.

Figure 2(a) shows the Raman spectra of the ALD compliant BL and MOCVD NL (henceforth, the terms “ALD compliant BL” and “MOCVD NL” refer to the PDA-treated GaN BL grown by RP-ALD and the PDA-treated GaN NL grown by MOCVD, respectively.) The Raman shift at approximately 565–568 cm^−1^ corresponds to the E2 phonon mode, which is sensitive to the amount of strain^25^, and the strain-free GaN E2 peak is located at 567.60 cm^−1^ (dashed line)^26^. The E2 peak of the MOCVD NL shifts 1.86 cm^−1^ in the negative direction with respect to the strain-free GaN, indicating the tensile strain in the MOCVD NL. In GaN/sapphire hetero-epitaxy, it is expected that GaN is subjected to biaxial compressive strain, because sapphire shrinks more than GaN during cooling down and the translational periodicity of GaN (rotated by 30° with respect to sapphire) is greater than that of sapphire^27^. In MOCVD, the growth of GaN NL primarily follows three-dimensional island growth (Volmer-Weber mode)^28^ and coalescence of the grains results in a high density of dislocations^29^. The strain resulting from defects such as dislocations is largely tensile^30^. Therefore, the observed tensile strain may mainly originate from the dislocations in the MOCVD NL. By contrast, the ALD compliant BL exhibited a Raman E2 peak at 567.55 cm^−1^, with only a slight Raman shift of −0.05 cm^−1^ with respect to the strain-free GaN. This low Raman shift indicates that the ALD compliant BL is near strain-free, suggesting that the defect density is greatly suppressed in the ALD compliant BL.

Figure 2(b–e) shows the two-dimensional micro-Raman mapping of the E2 peak of the ALD compliant BL and MOCVD NL, illustrating the spatial distributions of Raman shift ((b) and (c)) and intensity ((d) and (e)) cross an area of 100 μm × 100 μm. Figure 2(b,c) reveals that the ALD compliant BL is almost strain free, as indicated by average Raman shift of −0.05 cm^−1^ with an uniform distribution, whereas the MOCVD NL is tensile-strained, with an average Raman shift of −1.86 cm^−1^. This result indicates that the stress distribution in these two GaN layers is rather homogeneous. Figure 2(d) shows that the Raman intensity distribution of the ALD compliant BL is quite uniform, with a small variation coefficient (CV) of only 2.41%. However, the Raman intensity fluctuates greatly in the MOCVD NL, as indicated by the large CV of 65.2% shown in Fig. 2(e). The highly uniform distribution in Raman intensity indicates the high uniformity of the ALD compliant BL, which can be ascribed to the intrinsic benefit of the ALD as a result of the self-limiting growth. It should be noted from Fig. 1(b) that the ALD compliant BL exhibited a significant near-band-edge PL spectral peak at 3.410 eV (λ = 363.4 nm), whereas the near-band-edge PL from the MOCVD NL was observed at 3.398 eV (λ = 364.9 nm). It has been reported that tensile stress gives rise to a decrease in bandgap energy in the MOCVD NL^31^. Accordingly, compared with the MOCVD NL, a 12 meV blue-shift of the near-band-edge PL peak of the ALD compliant BL also reveals the relaxation of tensile strain in the ALD compliant BL.

### Structural characteristics of the GaN/sapphire interface

**Figure 3:**
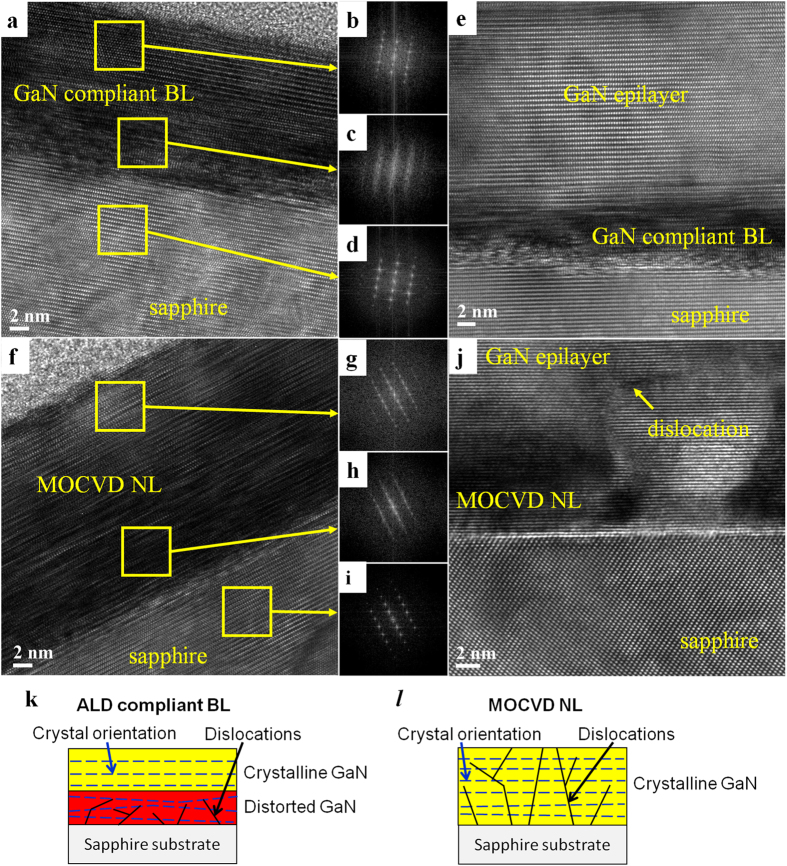
HRTEM images. (**a**,**e**) HRTEM images of the PDA-treated ALD compliant BL and sapphire substrate without the overlying GaN epilayer (**a**) and with the overlying GaN epilayer (**e**) grown by MOCVD. (**b**–**d**) FFT diffractograms of the areas enclosed in the upper and the bottom regions of the ALD compliant BL, and the sapphire in (**a**), respectively. (**f**,**j**) HRTEM images of the PDA-treated MOCVD NL and sapphire substrate without the overlying GaN epilayer (**f**) and with the overlying GaN epilayer (**j**) grown by MOCVD. A TD is indicated in (**j**), which was taken by a condition so as to excite exclusively the 0002 reflections and off any *hki*0 reflections of GaN. (**g**–**i**) FFT diffractograms of the areas enclosed in the upper and the bottom regions of the MOCVD NL, and the sapphire in (**f**), respectively. (**k**,**l**) show schematic diagrams of the ALD compliant BL and MOCVD NL, respectively.

Figure 3(a) displays a high-resolution transmission electron microscopy (HRTEM) image of the cross section including the ALD compliant BL and sapphire substrate. The fast Fourier transform (FFT) diffractograms shown in Fig. 3(b–d) refer to the areas enclosed in the upper region of the ALD compliant BL, the bottom region of the ALD compliant BL, and the sapphire substrate, respectively. The diffractograms in Fig. 3(b,d) show that a single crystal of GaN with the wurtzite structure grew with the following epitaxial relation with respect to the substrate: [0001] _GaN_ // [0001] _sapphire_ and 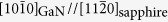. As shown in Fig. 1(a), the as-deposited GaN is composed of fine grains exhibiting the diffuse (0002) XRD peak. The coalescence and recrystallization of the fine grains caused by the PDA treatment lead to a nearly perfect single crystal with a strong (0002) XRD peak. Figure 3(e) shows an HRTEM image of the overlying GaN epilayer, the ALD compliant BL and sapphire substrate in the InGaN/GaN LED structure. As shown in Fig. 3(a,e), the lattice distortion can be observed in the BL, but it is only within ~10 nm from the interface. An influence of the substrate lattice is seen in this region near the interface where the GaN lattice is heavily distorted, which is clearly recognized by comparing the FFT diffractograms shown in Fig. 3(b,c). This heavily distorted GaN may absorb the misfit dislocations generated by the large lattice mismatch and thus prevent these dislocations from propagating into the overlying GaN epilayer, as shown schematically in Fig. 3(k). As a result, the strain caused by lattice misfit is relaxed within this heavily distorted region, leading to the near strain-free ALD compliant BL as revealed by the PL and Raman spectroscopy shown in Figs 1(b) and 2(a).

For reference, an HRTEM image of the cross section including the MOCVD NL and sapphire substrate is provided in Fig. 3(f). The HRTEM image and the corresponding FFT diffractogram of the upper region of MOCVD NL (Fig. 3(g)) indicate a single crystal GaN with the wurtzite structure, but the GaN has poor crystallinity compared to the upper region of the ALD compliant BL (which is consistent with the XRD measurement shown in Fig. 1(a)). The HRTEM image in Fig. 3(f) and the FFT diffractogram in Fig. 3(h) show that the lattice distortion in the bottom region of MOCVD NL is heavier than that in the upper region of MOCVD NL (Fig. 3(g)) but small compared with that of the bottom region of the ALD compliant BL (Fig. 3(c)). The MOCVD deposited layer grows epitaxially on top surface of the underlying layer (i.e., the substrate or the just as-deposited film). Therefore, all the (0002) lattice fringes appear parallel to the substrate surface from just the first layer along the GaN/sapphire interface, as shown in Fig. 3(j). The misfit dislocations are formed at the interface to accommodate the lattice mismatch, and then extend as TDs into the upper layer, as shown schematically in Fig. 3(l). An example of TDs is indicated in Fig. 3(j). The misfit dislocations formed at the interface and extended as TDs in the buffer layer and still into the upper layer. Hence, the lattice mismatch affects not only the MOCVD NL but also the entire InGaN/GaN LED structure. By contrast, the ALD compliant BL grew via the recrystallization and coalescence of the fine grains during the PDA treatment. Therefore, the formation of misfit dislocations was not intrinsic so that only a few dislocations were observed on the top of the buffer layer.

### Reduction in threading dislocation (TD) density in the GaN epilayer

**Figure 4:**
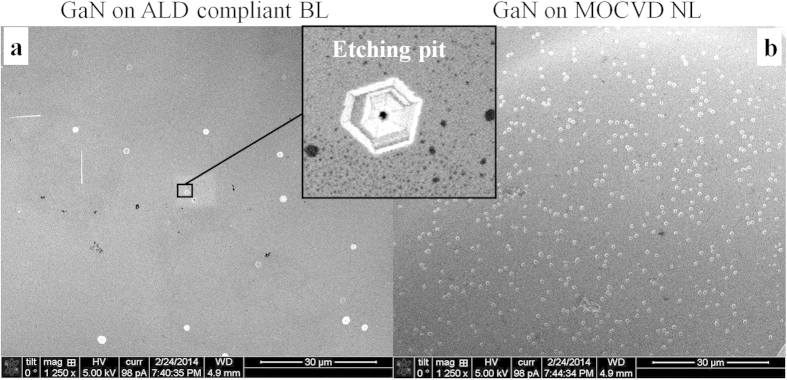
EPD measurement. SEM images of EPs on the top surface of the *n*-GaN layer in the InGaN/GaN LEDs grown on the ALD compliant BL (**a**) and MOCVD NL (**b**).

The etch-pit (EP) densities on the top surface of the *n*-GaN epilayer in the InGaN/GaN LEDs (without the metal contact) grown on the ALD compliant BL and MOCVD NL were obtained by counting the number of EP in the scanning electron microscope (SEM) images. Figure 4(a) displays that the EP density in the LED grown on the ALD compliant BL is as low as 2.2 × 10^5^ cm^−2^, which is much lower than that on the MOCVD NL shown in Fig. 4(b). Because each EP corresponds to a TD^32^, the result clearly indicates that TDs can be effectively suppressed by the ALD compliant BL. It should be noted that the TD density is typically very large, in the range of 10^7^–10^10^ cm^−2^, in the LEDs grown on sapphire substrate with the conventional MOCVD NL. In particular, this ultralow TD density is close to that reported in the thick GaN substrate grown by hydride vapor phase epitaxy (10^4^–10^6^ cm^−2^)^33^,^34^ and is approximately one order of magnitude lower than that typically obtained by the ELOG technique (10^6^–10^7^ cm^−2^)^13^,^14^. Hence, Fig. 4 provides clear evidence of a substantial TD reduction in the GaN epilayer by the ALD compliant BL.

**Figure 5:**
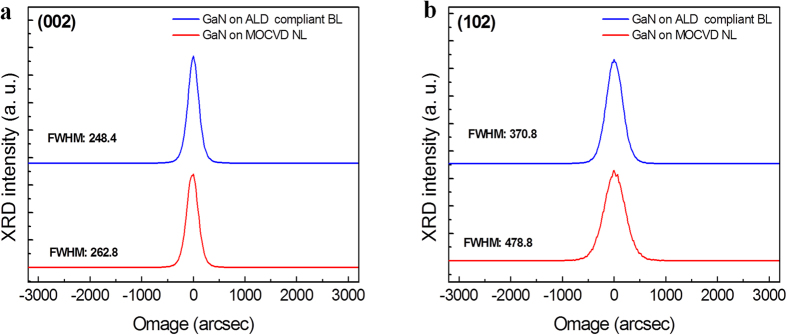
XRD of the GaN epilayers. XRD rocking curves of (**a**) the symmetrical (002) and (**b**) symmetrical (102) reflections of the GaN epilayers grown on the ALD compliant BL and MOCVD NL.

Figure 5 shows the (002) and (102) rocking curves of the GaN epilayers grown on ALD compliant BL and MOCVD NL, respectively. Both the (002) and (102) FWHM of the GaN on the ALD compliant BL (248.4 and 370.8 arcsec, respectively) are smaller than those of the GaN on the MOCVD NL (262.8 and 478.8 arcsec, respectively), which can be attributed to the decrease in TD density.

### Electrical and optical characteristics of the InGaN/GaN LEDs

**Figure 6:**
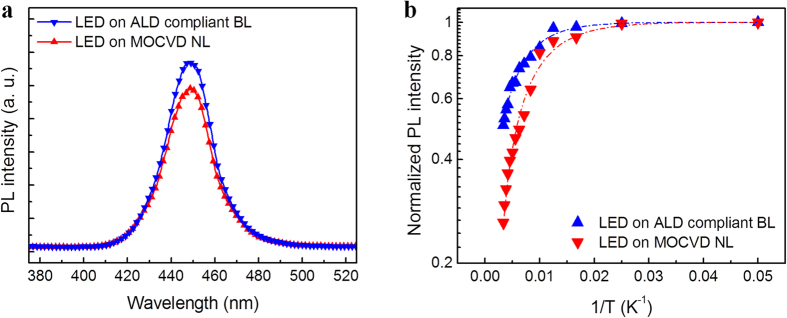
PL spectra and intensities of the InGaN/GaN LEDs. (**a**) Room-temperature PL spectra of LEDs on the ALD compliant BL and MOCVD NL. (**b**) Arrhenius plot of the normalized integrated PL intensity of the LEDs on the ALD compliant BL and MOCVD NL. The PL measurement was carried out at temperatures from 20 to 300 K. The dashed lines are the fitting curves described by the empirical Arrhenius equation defined in the Supplementary Information.

Figure 6 shows the room-temperature PL spectra and the temperature dependence of the normalized integrated PL intensity of the InGaN/GaN LEDs grown on the ALD compliant BL and MOCVD NL, respectively. As shown in Fig. 6(a), the wavelengths of the PL peak of both the LEDs grown on the ALD compliant BL and MOCVD NL are approximately the same (~449 nm), indicating that the indium composition of the InGaN MQWs in both the LEDs is identical. The peak PL intensity of the LED on the ALD compliant BL is higher than that on the MOCVD NL, which may be caused by the lower TD density due to the ALD compliant BL. The temperature dependence of the normalized integrated PL intensity, as shown in Fig. 6(b), can be described by the empirical Arrhenius equation (shown in Supplementary Information). The fitting curves give the activation energy *E*_*a*_ of 20.5 meV for the LED on the ALD compliant BL and 12.5 meV for the MOCVD NL. The higher activation energy *E*_*a*_ of nonradiative recombination centers is indicative of the superior light emission efficiency of the LED on the ALD compliant BL. The constant *C* (proportional to the density of nonradiative recombination centers) is 0.69 and 1.01 for the LEDs on the ALD compliant BL and MOCVD NL, respectively, indicating that a lower density of nonradiative recombination centers in the GaN grown on the ALD compliant BL. The result agrees well with the EP density shown in Fig. 4 because the TD is strongly associated with the nonradiative recombination centers in GaN^35^.

**Figure 7:**
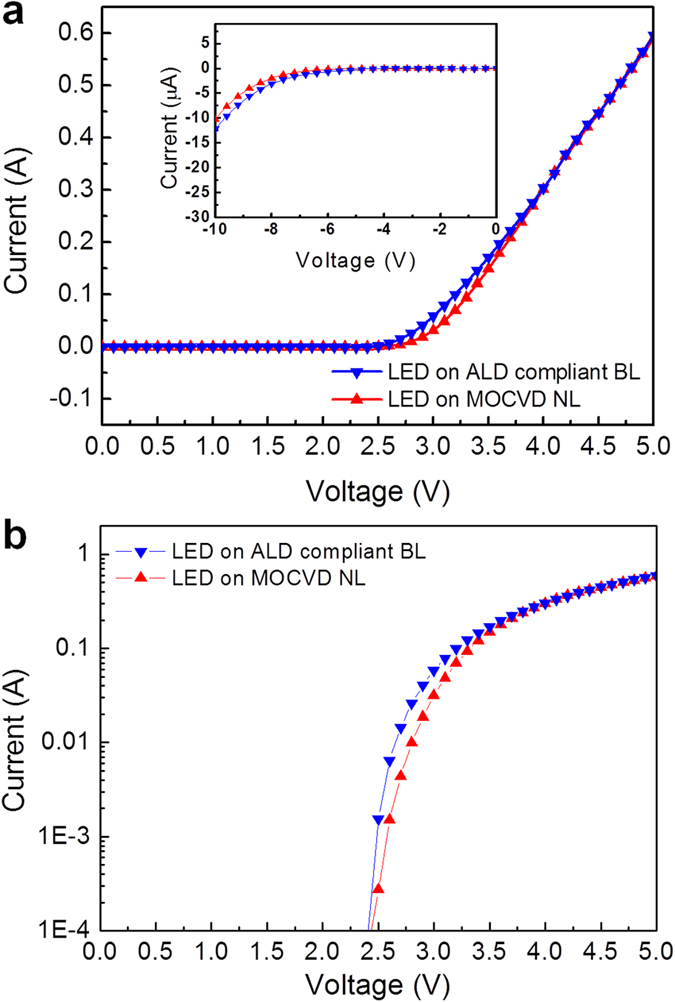
Electrical performances of the InGaN/GaN LEDs. (**a**) *I–V* curves of the InGaN/GaN LEDs grown on the ALD compliant BL and MOCVD NL. The inset shows the *I–V* characteristics of both the LEDs at reverse bias. (**b**) The forward biased *I–V* curves in the semi-long scale.

**Figure 8:**
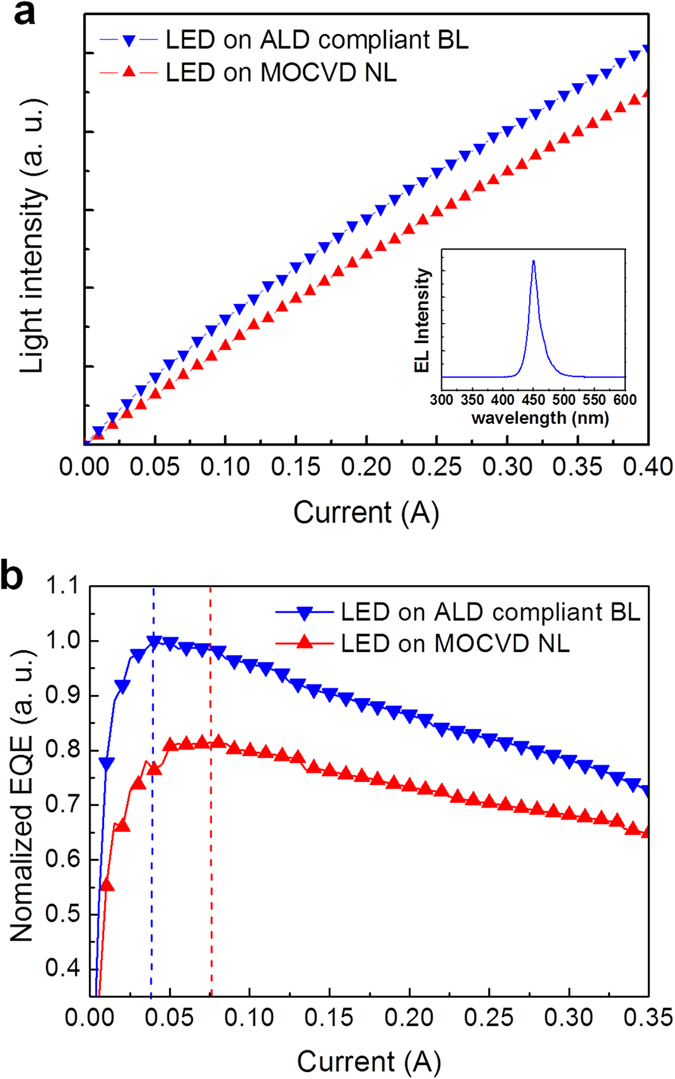
Optical performances of the InGaN/GaN LEDs. (**a**) EL intensity as a function of injection current from 1 to 400 mA of the InGaN/GaN LEDs grown on the ALD compliant BL and MOCVD NL. The inset represents a room-temperature EL spectrum of the LED grown on the ALD compliant BL. (**b**) The normalized EQE as a function of the injected current of the InGaN/GaN LEDs grown on the ALD compliant BL and MOCVD NL.

Figure 7(a,b) display the current vs. voltage (*I–V*) curves (in linear and semi-log scale, respectively), revealing the electrical characteristics of the InGaN/GaN LEDs grown on the ALD compliant BL and MOCVD NL. The forward voltages at the input current of 350 mA are found to be 4.149 and 4.151 V for the LEDs on the ALD compliant BL and MOCVD NL, respectively. The inset in Fig. 7(a) shows that the leakage currents at −10 V are approximately the same in both of the LEDs. Figure 8(a) presents the electroluminescence (EL) intensity as a function of the injection current of both the InGaN/GaN LEDs. The LED grown on the ALD compliant BL exhibited greater light emission efficiency, which could be attributed to the suppressed nonradiative recombination by the reduced TD density. The inset in Fig. 8(a) shows the EL spectrum of the LED grown on the ALD compliant BL, with a center peak at 450 nm originating from the InGaN/GaN MQWs. Table S1 in the Supplementary Information summarizes the electrical and optical properties of the InGaN/GaN LEDs grown on the ALD compliant BL and MOCVD NL. The reduced TD density of 2.2 × 10^5^ cm^−2^ due to the ALD compliant BL is responsible for the higher light emission efficiency in the InGaN/GaN LED.

Figure 8(b) shows the external quantum efficiency (EQE) as a function of the injection current of both the InGaN/GaN LEDs grown on the ALD compliant BL and MOCVD NL. Compared with the LED grown on the MOCVD NL, the EQE is higher, and the maximum EQE occurs at a lower injection current in the LED on the ALD compliant BL, which can be explained by the different Shockley-Read-Hall (SRH) nonradiative recombination rates. The higher EQE and the maximum EQE at a lower injection current (indicated by dashed lines) are attributed to the smaller SRH nonradiative recombination rate in the LED on the ALD compliant BL^36^,^37^, which is in very good agreement with the lower TD density, higher PL and EL intensity, and higher activation energy of nonradiative recombination centers of the LED on the ALD compliant BL as shown in Figs 4,6 and 8(a).

## Conclusion

In conclusion, high-quality hetero-epitaxial InGaN/GaN LEDs have been developed with an unusual TD density, as low as 2.2 × 10^5^ cm^−2^ on the ALD compliant BL. The GaN compliant BL grown by RP-ALD with subsequent PDA treatment was nearly free of strain, with uniform distribution, high crystalline quality, and a low defect density, which can be attributed to the intrinsic benefits of the self-limiting ALD growth and the recrystallization of small GaN grains by the PDA treatment. The misfit dislocations due to the large lattice mismatch were absorbed in a small region within ~10 nm from the interface, where the GaN lattices in the ALD compliant BL were heavily distorted, as shown in the HRTEM images and FFT diffractograms. Hence the strain caused by the lattice misfit is relaxed, and the dislocations are prevented from propagating into the overlying GaN epilayer. The EP density measurements indicate that the TD density in the overlying GaN epilayer was significantly suppressed by the near strain-free ALD compliant BL. As a result, the InGaN/GaN LED on the ALD compliant BL exhibited a higher activation energy of nonradiative recombination centers and a higher light emission efficiency, compared with that grown on the conventional MOCVD NL. Therefore, the near strain-free ALD compliant BL is a very promising growth template for large-area hetero-epitaxy in efficient LEDs and HEMTs for next-generation high-efficiency solid-state lighting and high-power electronics.

## Methods

### ALD compliant BL

A GaN compliant BL of ~20 nm thickness was deposited on the (0001)-oriented sapphire substrate at 500 °C. The growth was carried out in an ALD reactor with the remote plasma configuration, which can minimize the plasma-induced damages on the deposited films. Triethylgallium (TEGa, Ga(C_2_H_5_)_3_) and nitrogen radicals were the precursors for gallium and nitrogen, respectively. The remote nitrogen radicals were generated by a radio-frequency coil under the injection of NH_3_/H_2_ pulses. Each ALD cycle comprises the following steps: TEGa pulse → Ar purge → remote NH_3_/H_2_ plasma → Ar purge, as shown schematically in Figure S1 in the Supplementary Information. After these steps, the GaN BL was treated by PDA at 1130°C to improve its crystallinity.

### InGaN/GaN LED

The LED structure was grown on the ALD compliant BL by an atmospheric-pressure MOCVD system. Figure S4 in the Supplementary Information shows the schematic diagram of the LED structure, which consists of 2.5 μm unintentionally doped GaN, 3 μm Si-doped *n*-type GaN, an unintentionally doped active layer with 5 pairs of In_*x*_Ga_1−*x*_N/GaN MQWs (0.15 < *x* < 0.18), and 100 nm Mg doped *p*-type GaN. The LEDs with an active area of 1 × 1 mm^2^ were formed by conventional photolithography. TiAlNiAu was used as the Ohmic contact to *n*-type GaN and NiAu to *p*-type GaN. For comparison, reference LEDs with a similar multilayer structure were also fabricated. All of the thickness and growth conditions of each layer in the reference LED are the same as those shown in Figure S4, except that the ALD compliant BL was replaced by the MOCVD NL with a GaN thickness of ~25 nm grown by MOCVD at 500 °C. The conditions of PDA treatment on the MOCVD NL were the same as those on the ALD compliant BL.

### Measurements

The thickness of the GaN BL was measured by a spectroscopic ellipsometer in the spectral range between 300 and 1000 nm at an incident angle of 70°. The crystalline phase and quality of the ALD compliant BL and MOCVD NL were characterized with HRTEM (FEI Tecnai G2 F20) as well as a high-power X-ray diffractometer (Rigaku TTRAX 3, 18 kW). The EPs on top surface of the *n*-type GaN epilayer were generated by chemical etching in a H_3_PO_4_ solution at 250 °C for 15 min, and the EP densities were calculated by counting the number of EP in the images taken by SEM (FEI Dual-Beam NOVA 600i). The optical properties of the ALD compliant BL and MOCVD NL were characterized by micro-Raman and -PL spectroscopy (Uni-RAM). The samples were excited by an argon laser (λ = 488 nm, continuous wave) and a pulsed Q-switched diode-pumped solid-state laser (λ = 266 nm, repetition rate = 3 kHz) for the Raman and PL measurements, respectively. The PL and Raman spectra were collected by a microscope and a spectrograph (Andor Shamrock SR-500i) equipped with a thermoelectrically cooled Andor-iDus CCD of 1024 × 256 pixels. Raman mapping was carried out by the SigmaKoki motorized stage with a spatial resolution of 1 μm. The temperature-dependent PL measurement was performed at the temperatures from 20 to 300 K using a He-Cd laser (λ = 325 nm) as the excitation source. The *I–V* characteristics of the LEDs were measured using an Agilent B1500A semiconductor parameter analyzer. The EL intensities and spectra of the LEDs were measured with an Ocean USB4000 spectrometer at room temperature. The EQE was measured from the packaged LEDs in an integrating sphere at room temperature under DC operation.

## Additional Information

**How to cite this article**: Shih, H.-Y. *et al.* Ultralow threading dislocation density in GaN epilayer on near-strain-free GaN compliant buffer layer and its applications in hetero-epitaxial LEDs. *Sci. Rep.* **5**, 13671; doi: 10.1038/srep13671 (2015).

## Supplementary Material

Supplementary Information
