# Supplementary material for: Ultralow threading dislocation density in GaN epilayer on near-strain-free GaN compliant buffer layer and its applications in hetero-epitaxial LEDs
Source: Sci Rep. 2015 Sep 2;5:13671. doi: 10.1038/srep13671 (PMC4556983; doi:10.1038/srep13671)
Supplement: Supplementary Information [file srep13671-s1.doc]

**Ultralow threading dislocation density in GaN epilayer on near-strain-free GaN compliant buffer layer and its applications in hetero-epitaxial LEDs**

**(Supplementary Information)**

*Huan-Yu Shih^a^, Makoto Shiojiri^b^, Ching-Hsiang Chen^c^, Sheng-Fu Yu^d^, Chung-Ting Ko^a^*, *Jer-Ren Yang^a^, Ray-Ming Lin^d^,**, *Miin-Jang Chen^a^,**

^a^ Department of Materials Science and Engineering, National Taiwan University, Taipei, Taiwan

^b^ Kyoto Institute of Technology, Kyoto, Japan

^c^ Graduate Institute of Applied Science & Technology, National Taiwan University of Science & Technology, Taipei, Taiwan

^d^ Department of Electronic Engineering, Chang Gung University, Taoyuan, Taiwan

*^*^* Authors to whom correspondence should be addressed.

E-mail: rmlin@mail.cgu.edu.tw and mjchen@ntu.edu.tw

Phone: +886(0)32118800 #5790 and +886(0)233665301

**Process and self-limiting characteristics of ALD GaN**

**
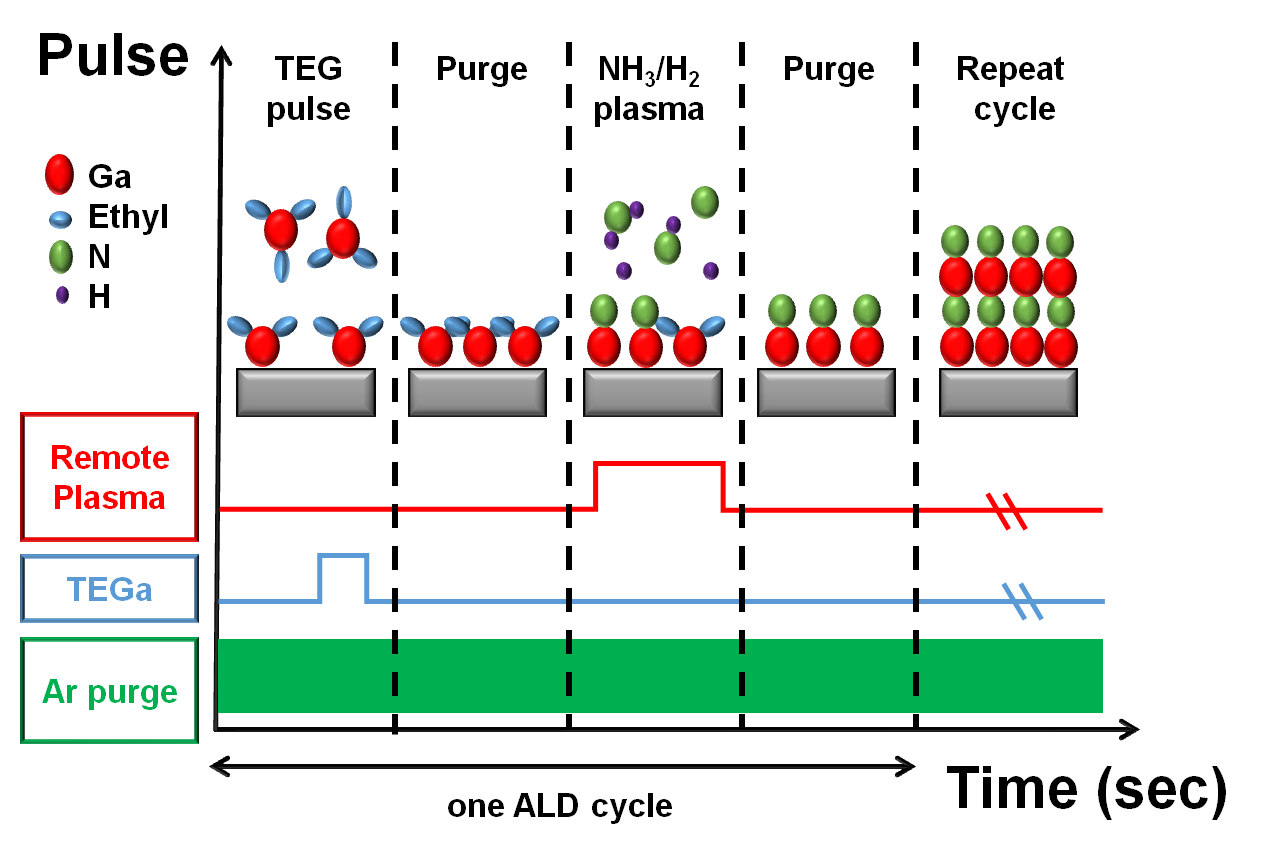
**

**Figure S1** PR-ALD process for growing the GaN BL on the (0001) sapphire substrate.


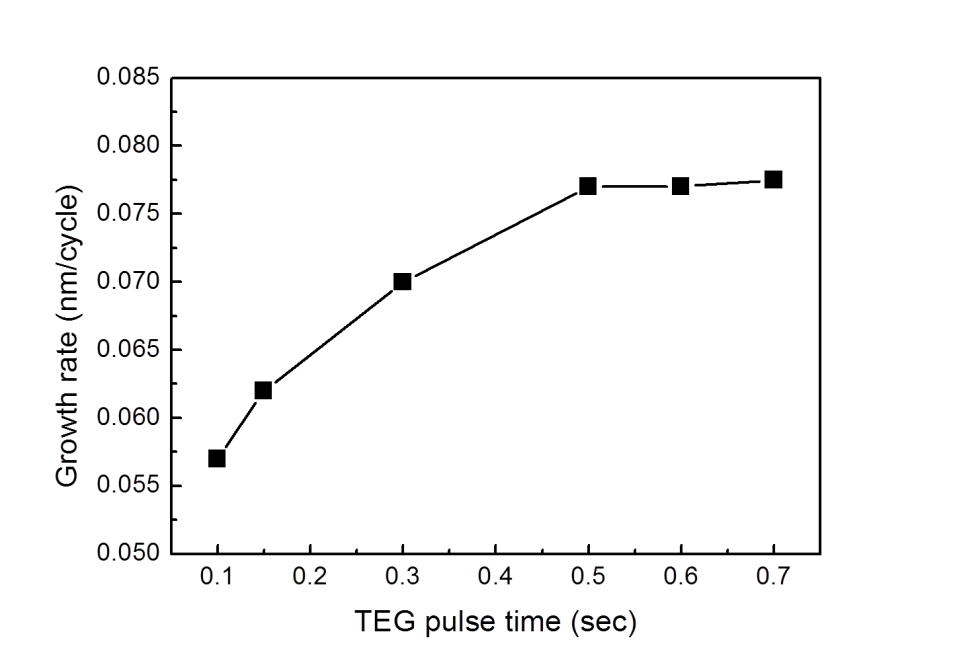


**Figure S2**  Dependence of GaN growth rate per ALD cycle on the TEG pulse time. The growth rate increases with the TEG dose and then saturates at ~0.077 nm/cycle when the TEG pulse time is greater than 0.5 sec, suggesting that the growth of GaN exhibited the self-limiting characteristics.


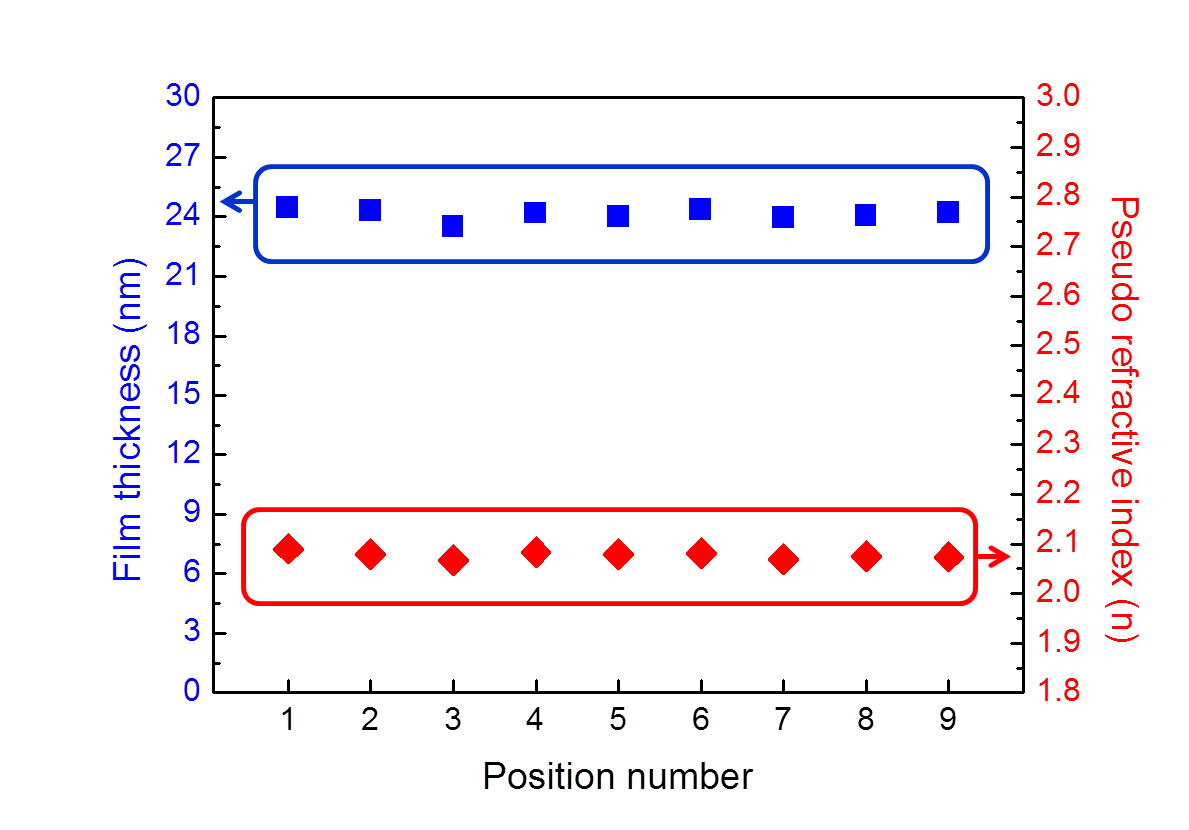


**Figure S3** The film thickness and the pseudo-refractive index of the as-deposited GaN BL for different points on sapphire substrate with an effective area of 6-inch diameter. The positions #1~#9 were taken radially from the center with a spacing of ~1.5 inch. The pseudo refractive index was extracted by the spectroscopic ellipsometer at λ= 633 nm from the direct measurement on
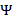
 and
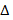
(the amplitude ratio and phase difference between *p*- and *s*-polarized light waves), based on an optical model which assumes a perfectly flat substrate with infinite thickness [1].

**Structure of the InGaN/GaN LED**

**
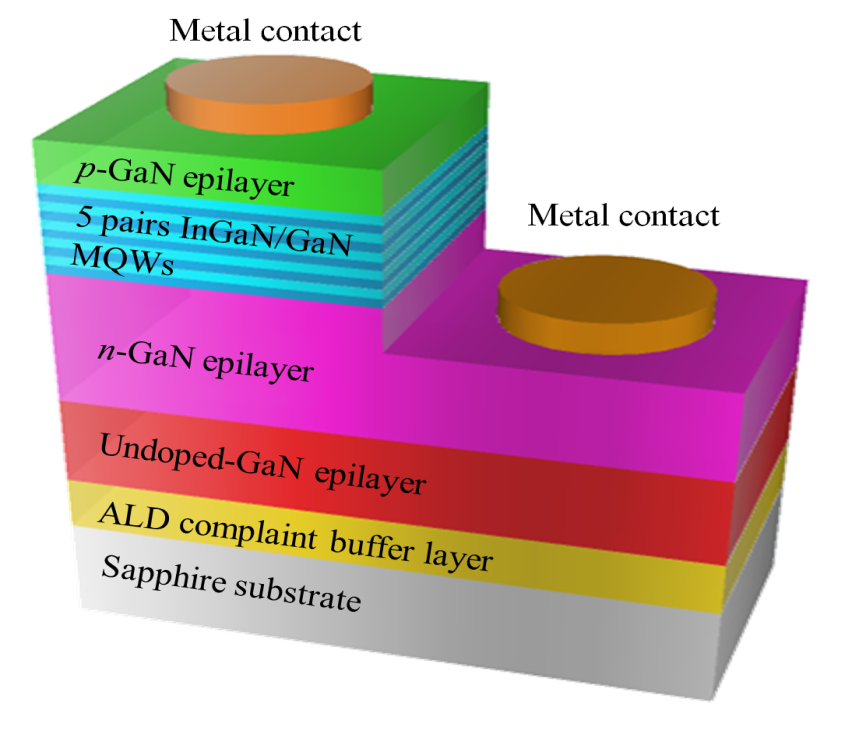
**

**Figure S4** Schematic structure of the InGaN/GaN LED grown on the ALD complaint BL.

**Optical properties of the GaN BL grown by RP-ALD**

The refractive index and extinction coefficient of the as-deposited GaN BL grown by RP-ALD were investigated by the spectroscopic ellipsometry (SE). Figure S5 shows the dispersion of the refractive index and extinction coefficient in the wavelength range between 300 and 1000 nm. The Tauc-Lorentz model, which is widely used for the amorphous and polycrystalline semiconductors, was used to fit the SE data^1^. The refractive index of the as-deposited GaN BL at λ=633 nm is ~2.17 and the bandgap energy deduced from the extinction coefficient is ~3.48 eV, close to the reported values of GaN thin films^2^.


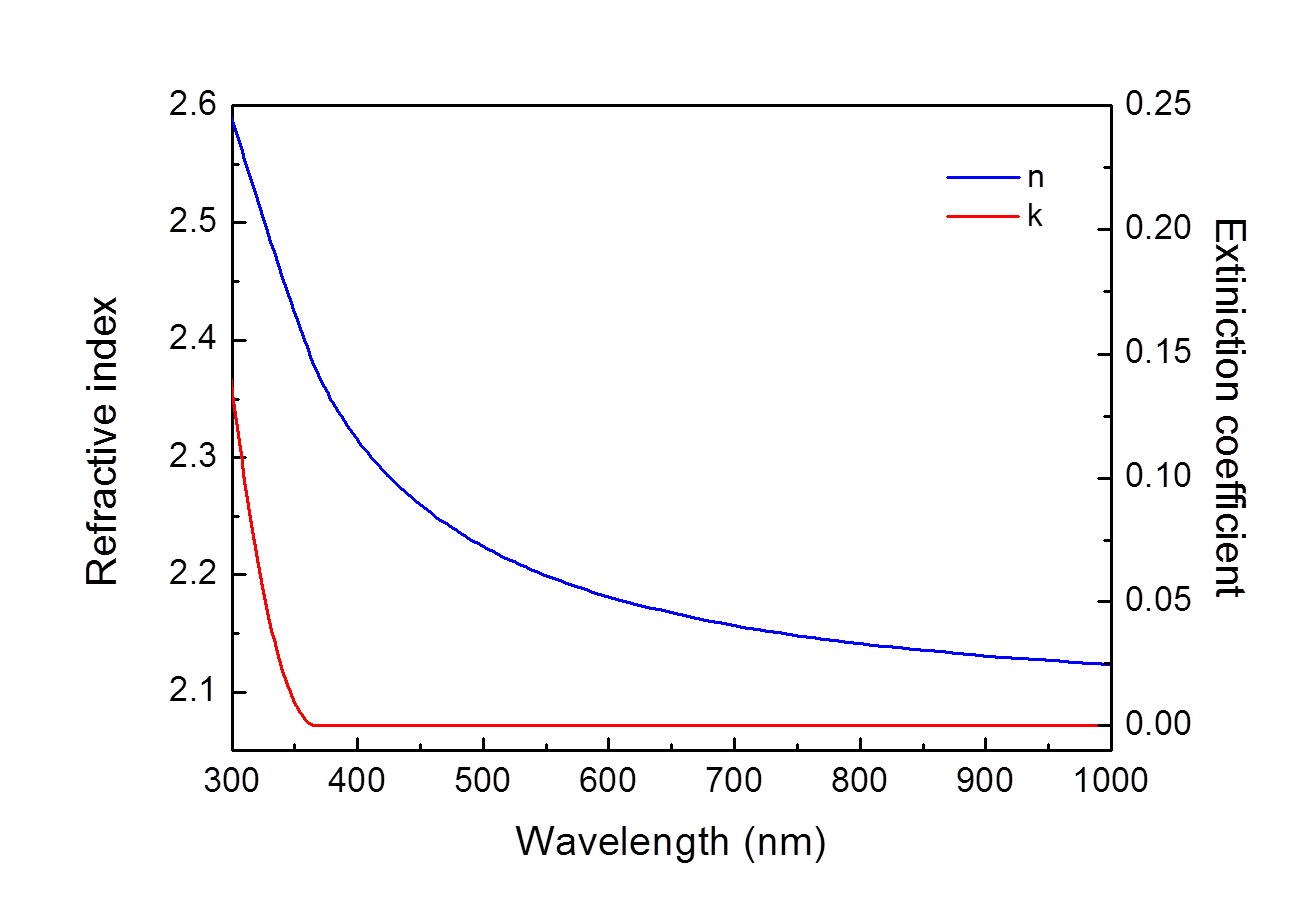


**Figure S5** The refractive index and extinction coefficient as a function of wavelength of the as-deposited GaN BL grown by RP-ALD.

**Empirical Arrhenius equation**

A simple physical mode used to fit the Arrhenius plot of the integrated PL intensity is given as follows^3^:

$$I(T)=\frac{I_{0}}{1+C\exp\left(\frac{E_{a}}{k_{B}T}\right)}$$

where *I_0_* is the PL intensity at low temperature, *C* is a constant proportional to the density of nonradiative recombination centers, *E_a_* is the activation energy of nonradiative recombination centers, *T* is the temperature, and *k_B_* is the Boltzmann constant.

**Summary of the electrical and optical properties of the InGaN/GaN LEDs grown on the ALD compliant BL and MOCVD NL**

**Table S1**

|  | Leakage current at -10V | Forward voltage at 350 mA | Relative EL intensity at 350 mA (a.u.) |
| --- | --- | --- | --- |
| ALD compliant BL | -12.19 μA | 4.149 V | 9.140×10^-6^ |
| MOCVD NL | -10.25 μA | 4.151 V | 7.985×10^-6^ |

Reference

1 Fujiwara, H. *Spectroscopic ellipsometry : principles and applications*. (John Wiley, 2007).

2 El-Naggar, A. M., Ei-Zaiat, S. Y. & Hassan, S. M. Optical parameters of epitaxial GaN thin film on Si substrate from the reflection spectrum. *Opt Laser Technol* **41**, 334-338; DOI: 10.1016/j.optlastec.2008.05.022 (2009).

3 Teo, K. L. *et al.* An analysis of temperature dependent photoluminescence line shapes in InGaN. *Appl Phys Lett* **73**, 1697-1699; DOI: 10.1063/1.122249 (1998).
